# Physical activity and academic burnout among middle school students: uncovering cognitive reappraisal and expressive suppression

**DOI:** 10.3389/fpsyg.2026.1780820

**Published:** 2026-06-15

**Authors:** Jiaxi Chen, Linghui Zou, Ziping Fan, Xiu Feng, Xinbo Wu, Dianhui Peng, Weixin Dong, Chunxia Lu, Jun Chen

**Affiliations:** Department of Sport Education, Hunan Normal University, Changsha, Hunan, China

**Keywords:** academic burnout, cognitive reappraisal, expressive suppression, inconsistent indirect effect, physical activity

## Abstract

**Objective:**

This study examined whether cognitive reappraisal and expressive suppression mediate the association between physical activity and academic burnout among students at Huaihua Experimental Junior High School.

**Methods:**

A cross-sectional study was conducted among 1,529 junior high school students. Participants completed the Learning Burnout Scale for Middle School Students (LBS-MSS), the Emotion Regulation Questionnaire for Children and Adolescents (ERQ-CA), and the Physical Activity Rating Scale-3 (PARS-3). Data were analyzed using SPSS 28.0, Amos 27.0, and the PROCESS macro.

**Results:**

Academic burnout was negatively associated with physical activity (*r* = −0.279, *p* < 0.01), cognitive reappraisal (*r* = −0.267, *p* < 0.01), and expressive suppression (*r* = −0.060, *p* < 0.05). Physical activity was positively associated with cognitive reappraisal (*r* = 0.493, *p* < 0.01) and expressive suppression (*r* = 0.503, *p* < 0.01). Regression analysis showed that physical activity significantly predicted academic burnout (*β* = −0.131, *p* < 0.001). Mediation analyses indicated that cognitive reappraisal significantly mediated this association (indirect effect = −0.084), whereas expressive suppression showed an attenuating indirect effect (effect = 0.056).

**Conclusion:**

Physical activity was negatively associated with academic burnout among junior high school students. Cognitive reappraisal partially mediated this association, whereas expressive suppression showed an inconsistent indirect effect that attenuated part of the protective association. Given the modest effect sizes, these findings should be interpreted cautiously but suggest that different emotion regulation strategies may play distinct roles in the link between physical activity and academic burnout.

## Introduction

According to *the Outline of the Education Powerhouse Construction Plan (2024–2035)* (Central Committee of the Communist Party of China and State Council of the People’s Republic of China, 2025), China’s recent educational reform has increasingly emphasized students’ holistic development, including academic achievement and physical and mental health. In this context, academic burnout among junior high school students is an important concern, as this developmental stage involves rapid physical, cognitive, and emotional changes alongside intensified academic demands related to the high school entrance examination (Haritay et al., 2025).

Academic burnout (AB) is a psychological syndrome induced by chronic and excessive academic stress, characterized by core manifestations of emotional exhaustion, learning cynicism, and diminished academic efficacy (Liu Y. et al., 2024). For junior high school students, persistent burnout not only directly leads to decreased academic engagement (Martin-Rodriguez et al., 2024), declining performance, and avoidance behaviors but, on a deeper level, also erodes psychological capital such as intrinsic learning motivation and self-efficacy (Liu W. et al., 2024). It often forms a vicious cycle with emotional issues like anxiety and depression, posing a severe threat to their immediate healthy development and long-term life trajectories (Lee et al., 2025). Consequently, at the intersection of policy vision and real-world challenges, investigating effective strategies for preventing and intervening in academic burnout carries important practical significance.

Physical activity (PA) has received increasing attention as a modifiable factor that may promote adolescent mental health. Existing evidence suggests that regular physical activity is associated with reduced stress and anxiety, enhanced psychological well-being, and lower levels of academic burnout, while also contributing to greater academic engagement, self-efficacy, and stress resilience (Kang et al., 2024; Kolb et al., 2025; Latino et al., 2025). Emerging findings further indicate that physical activity is negatively associated with academic burnout among vulnerable youth populations, including rural left-behind children (Chen et al., 2025).

However, most existing studies have primarily focused on the direct relationship between physical activity and academic burnout, while the underlying psychological mechanisms remain insufficiently understood. Emotion regulation may represent one important explanatory pathway. According to Gross’s model (Gross, 1998), cognitive reappraisal (CR) and expressive suppression (ES) are two core emotion regulation strategies that may function through distinct pathways in this association. Cognitive reappraisal refers to reinterpreting stressful situations in a more adaptive way and is generally associated with better psychological adjustment. In contrast, expressive suppression refers to inhibiting outward emotional expression and has often been linked to greater psychological costs under stress. Therefore, these strategies may play different roles in the relationship between physical activity and academic burnout.

Given these theoretical and empirical considerations, it is necessary to further clarify how emotion regulation strategies may explain or modify the relationship between physical activity and academic burnout. Drawing on prior empirical evidence and relevant theoretical perspectives, this study examined the association between physical activity and academic burnout among junior high school students, with particular attention to the potential mediating roles of cognitive reappraisal and expressive suppression. Accordingly, the following hypotheses were proposed:

Hypothesis 1: Physical activity is negatively associated with academic burnout among junior high school students.

Hypothesis 2: Cognitive reappraisal would mediate the relationship between physical activity and academic burnout through a beneficial indirect pathway.

Hypothesis 3: Expressive suppression showed an attenuating indirect mediating effect in the relationship between physical activity and academic burnout.

**Figure 1:**
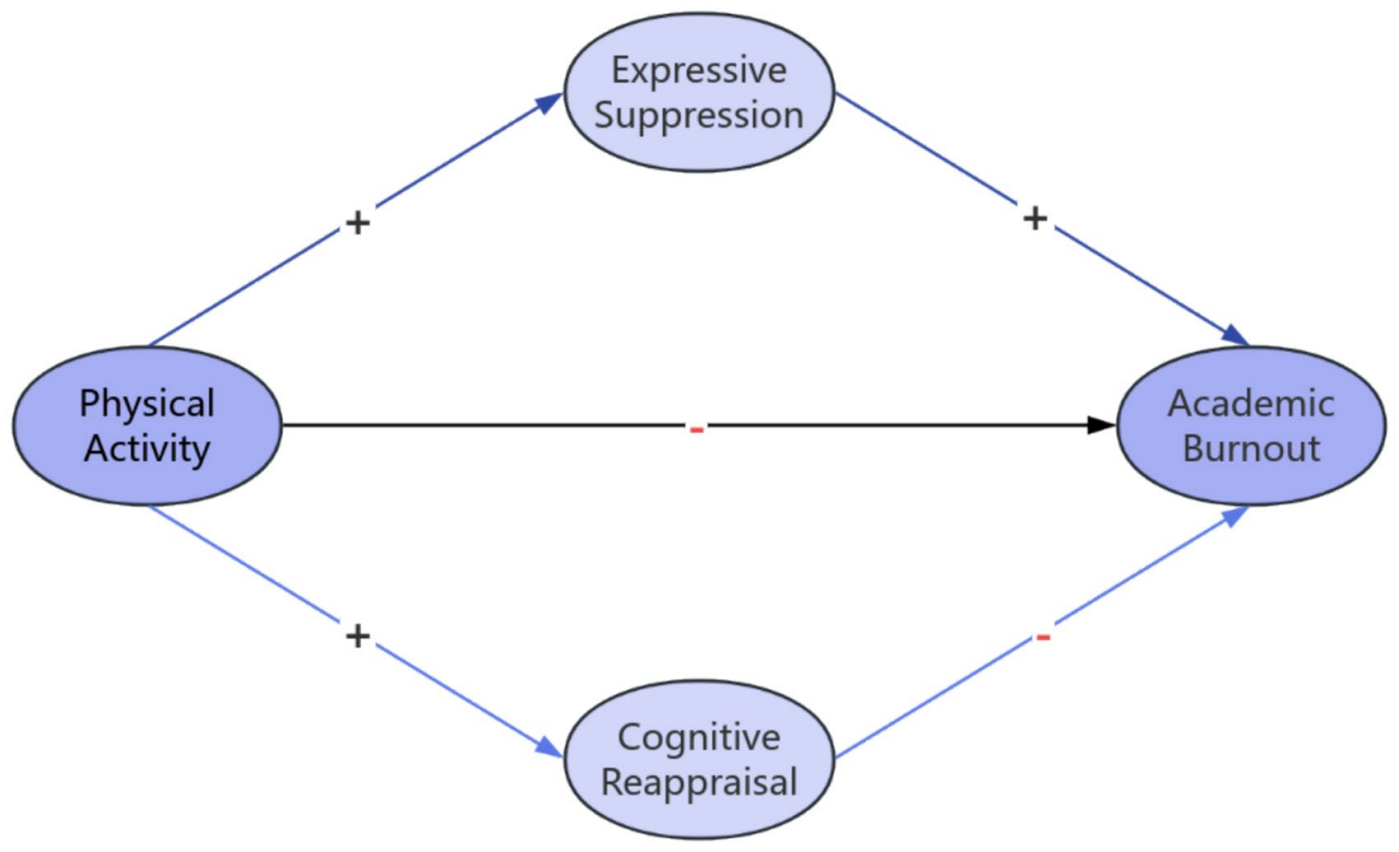
Hypothesized research model.

The proposed model is illustrated in Figure 1.

## Methods

### Study design

This study employed a cross-sectional design to systematically examine the relationships among physical activity, emotion regulation strategies (cognitive reappraisal and expressive suppression), and academic burnout among junior high school students.

### Participants and setting

From March to May 2023, cluster sampling was used to recruit students from Huaihua Experimental Middle School in Hunan Province, China. The sample included students from eight Grade 7 classes, six Grade 8 classes, and 11 Grade 9 classes.

### Data collection procedures

Prior to data collection, ethical approval was obtained, and written informed consent was acquired from school authorities, teachers, participants, and their legal guardians. Data collection was administered in the school computer laboratory. Participants completed the questionnaires electronically via an encrypted online survey platform under the direct supervision of researchers. The questionnaire comprised demographic information and three standardized measurement scales. Participation was voluntary, and all responses were anonymized to ensure confidentiality. After completion, data were exported directly from the survey platform and systematically screened for completeness and logical consistency to ensure data quality. The questionnaire consisted of two sections: demographic information (gender, age, grade, household registration type, sibling status, parental marital status, and guardian type) and standardized measures of physical activity, academic burnout, and emotion regulation. Participation was voluntary, and all responses were anonymized prior to analysis. Questionnaire data were exported directly from the platform and screened for completeness and consistency.

### Measures

#### Physical activity scale

Physical activity was assessed using the Chinese version of the Physical Activity Rating Scale (PARS-3), originally developed by Masao Hashimoto and subsequently translated and culturally adapted by Liang (1994). The PARS-3 is a self-report instrument consisting of three items that assess the intensity, frequency, and duration of physical activity over the past month. Each item is rated on a 5-point Likert scale ranging from 1 to 5. Total scores are calculated as Intensity × (Duration − 1) × Frequency, yielding a range from 0 to 100, with higher scores indicating greater levels of physical activity.

#### Academic burnout scale

Academic burnout was measured using the Learning Burnout Scale for Middle School Students (LBS-MSS), developed by Hu and Dai (2007). The scale consists of 21 items across four dimensions: Emotional Exhaustion (EE; 8 items), Alienation to Teachers (ATT; 4 items), Physical Exhaustion (PE; 4 items), and Reduced Learning Efficacy (RLE; 5 items, reverse-scored). All items are rated on a 5-point Likert scale ranging from 1 to 5. Higher total scores indicate higher levels of academic burnout.

#### Emotion regulation strategies scale

Emotion regulation strategies were assessed using the Emotion Regulation Questionnaire for Children and Adolescents (ERQ-CA). This instrument was revised by Chen et al. (2016) based on the original version developed by Gullone and Taffe (2012). The scale consists of 10 items measuring two dimensions: cognitive reappraisal (CR) and expressive suppression (ES). Items are rated on a 5-point Likert scale ranging from 1 (strongly disagree) to 5 (strongly agree). Higher subscale scores indicate greater use of the corresponding emotion regulation strategy.

### Data analysis

SPSS 28.0 was used for descriptive statistical analyses. Spearman correlation analyses were conducted for ordinal variables or variables that did not meet normality assumptions, whereas Pearson correlation analyses were used to examine the associations among physical activity, cognitive reappraisal, expressive suppression, and academic burnout. Structural equation modeling was performed using AMOS 27.0. Mediation analyses were conducted using Model 4 of the PROCESS macro (version 4.2) developed by Preacher and Hayes (2004). Cognitive reappraisal was tested as a mediating variable in the relationship between physical activity and academic burnout, whereas expressive suppression was examined as an inconsistent indirect pathway within the same association. Based on prior literature and demographic relevance, gender, age, grade, household registration type, sibling status, parental marital status, and guardian type were included as covariates. By adjusting for these factors, we aimed to reduce potential confounding influences and improve the robustness of the observed associations. Indirect effects were tested using a bootstrap procedure with 5,000 resamples. A 95% confidence interval that did not include zero was considered statistically significant. Statistical significance was set at *p* < 0.05, ^*^p < 0.01, and ^**^p < 0.001.

## Results

### Reliability and validity analysis of the questionnaires

All scales demonstrated acceptable internal consistency in the present sample. Cronbach’s *α* was 0.876 for the PARS-3, 0.895 for the LBS-MSS, and 0.865 for the ERQ-CA. For the ERQ-CA subscales, Cronbach’s *α* was 0.912 for cognitive reappraisal and 0.797 for expressive suppression. In addition, the Kaiser–Meyer–Olkin (KMO) values and Bartlett’s tests of sphericity supported the adequacy of the measurement instruments.

### Participant characteristics

A total of 1,529 valid questionnaires were retained for analysis, yielding a valid response rate of 99.54%. The final sample consisted of 786 boys (51.40%) and 743 girls (48.60%), with a mean age of 14.34 years (SD = 0.96). Grade distribution was as follows: 555 students (36.20%) in Grade 7,439 (28.70%) in Grade 8, and 535 (34.90%) in Grade 9. Detailed sociodemographic characteristics are presented in Table 1.

**Table 1:** Demographic characteristics of the participants (*N* = 1,529).

Variables	Categories	Frequency	Percentage (%)
Gender	Male	786	51.4
	Female	743	48.5
Age	12	1	0.1
	13	356	23.2
	14	467	30.5
	15	545	35.6
	16	152	9.9
	17	8	0.5
Grade	7	555	36.2
	8	439	28.7
	9	535	34.9
Household registration type	Rural *hukou*	848	55.4
	Urban *hukou*	681	44.5
Singleton status	Singleton	296	19.3
	Non-only-child	1,233	80.6
Parental marital status	Married	1,309	85.6
	Divorced	153	10.0
	Widow/Widower	19	1.2
	Remarried	48	3.1
Guardian type	Father or mother	1,373	89.7
	Paternal grandparents	84	5.4
	Maternal grandparents	22	1.4
	Others	50	3.2

### Common method bias

The common method bias test was conducted by Harman single factor test. A total of 7 factors with eigenvalues greater than 1 were extracted from the unrotated exploratory factor analysis results, and the maximum factor variance explanation rate was 24.564%, which was less than 40%. Therefore, no serious common method bias existed in this study.

### Correlation analysis of main variables

**Figure 2:**
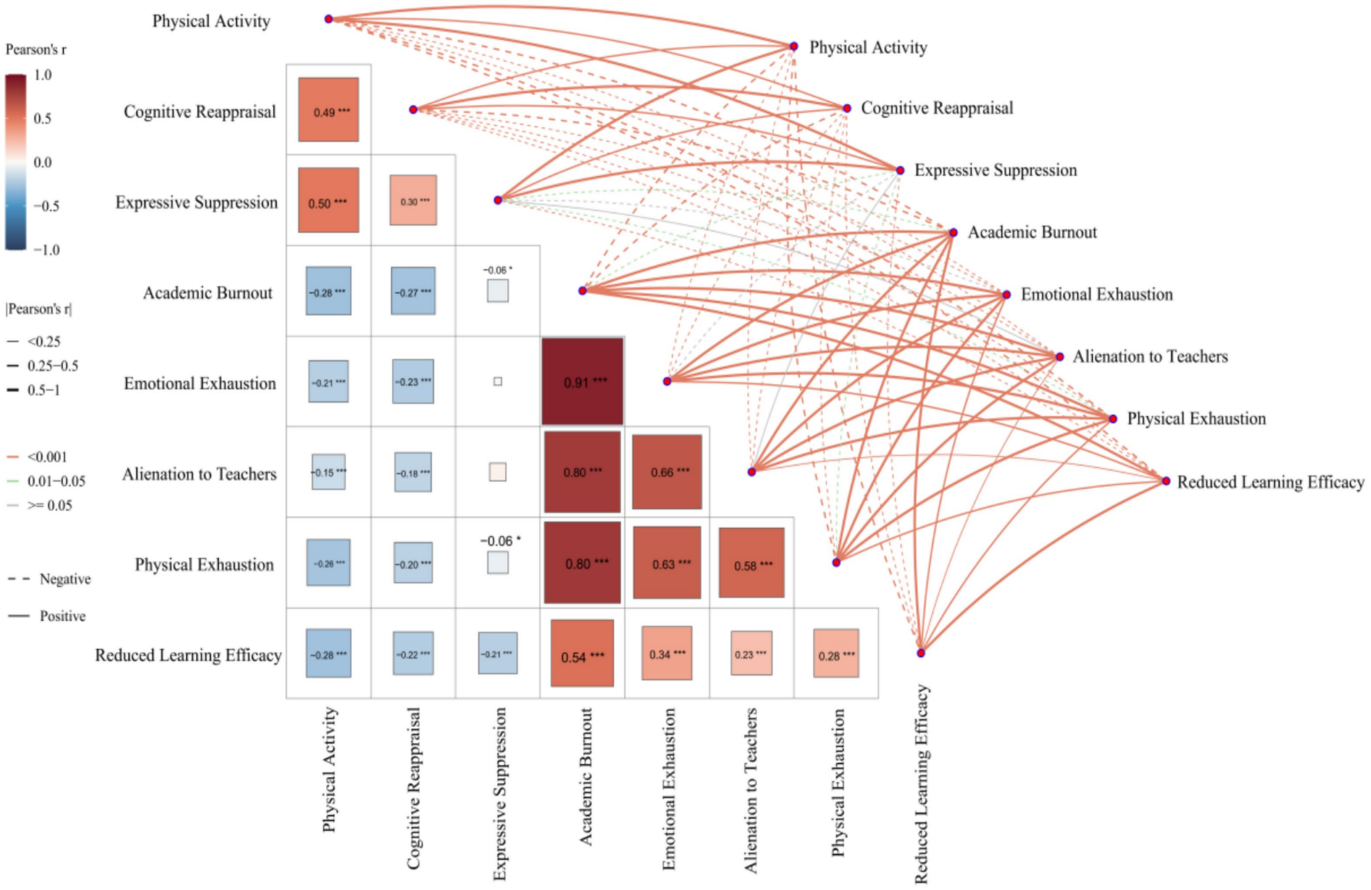
Correlation matrix among study variables.

The mean value, standard deviation and correlation coefficient of each variable were analyzed. The results showed that physical activity, cognitive reappraisal, expressive suppression and academic burnout were significantly correlated with each other (*p* < 0.05). In addition, sex and age were also correlated with predictive variables (*p* < 0.05), while other additional variables (such as household registration type, singleton status, parental marital status, and type of guardian, etc.) were not correlated with predictive variables. Although household registration type, singleton status, parental marital status, and type of guardian were not directly correlated with the main variables, they might still affect academic burnout in other ways. These factors could be considered in future research to explore potential indirect or moderating effects (see Figure 2).

### Hypothesis testing

#### Model overview

**Figure 3:**
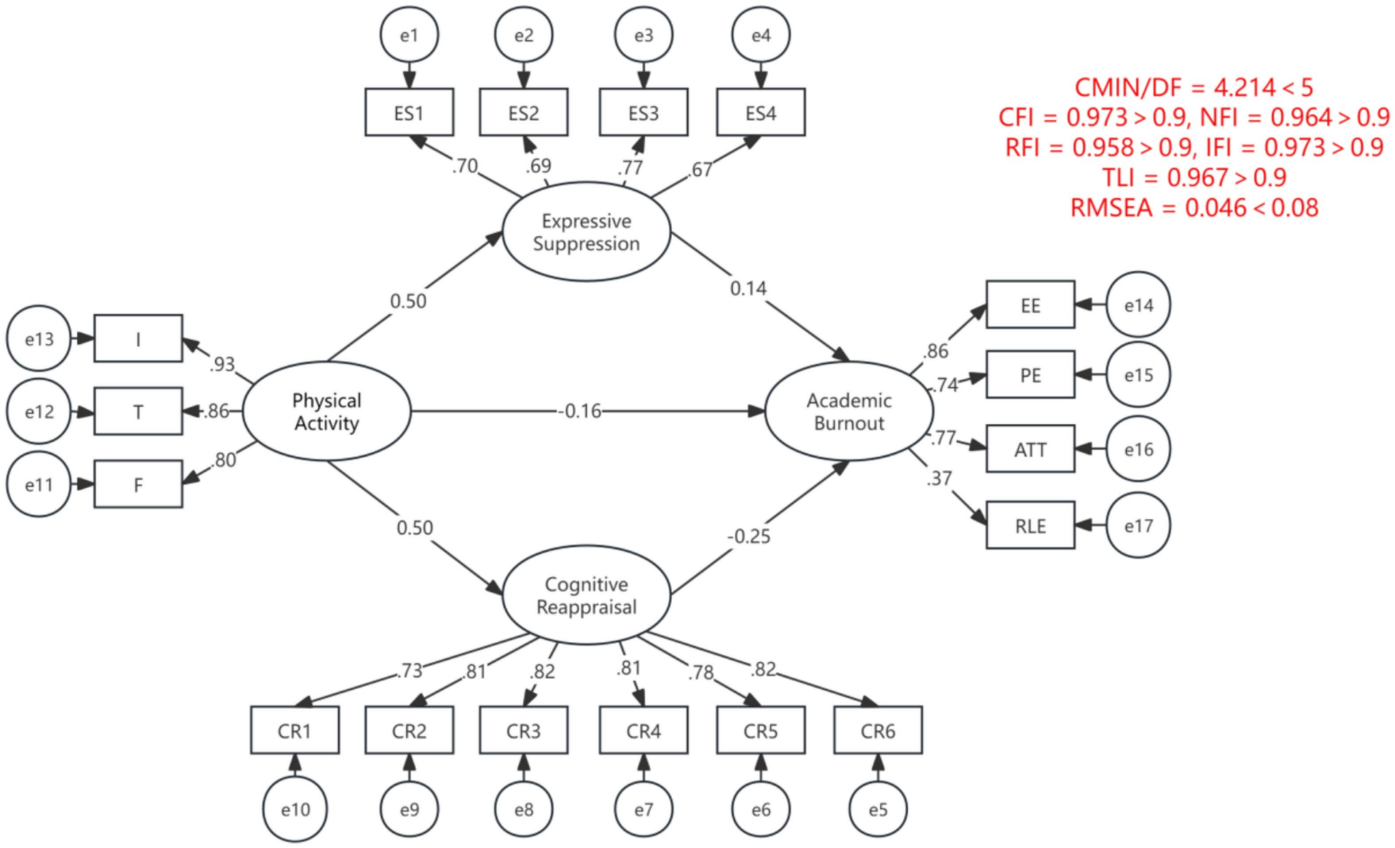
Structural modeling diagram between cognitive reappraisal, expressive suppression, physical activity, and academic burnout.

Physical activity was specified as the exogenous variable, academic burnout as the endogenous variable, and cognitive reappraisal and expressive suppression as parallel mediators. The model demonstrated acceptable fit to the data: CMIN/DF = 4.214, CFI = 0.973, NFI = 0.964, RFI = 0.958, IFI = 0.973, TLI = 0.967, RMSEA = 0.046. All fit indices met conventional thresholds for acceptable model fit (CMIN/DF < 5, CFI/NFI/RFI/IFI/TLI > 0.90, RMSEA < 0.08) (see Figure 3).

#### Direct effects

**Figure 4:**
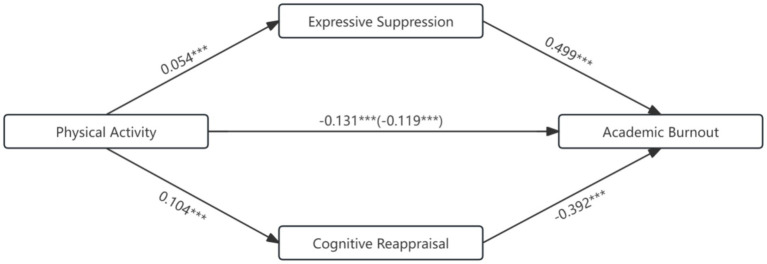
Mediating and inconsistent indirect effects of cognitive reappraisal and expressive suppression in the relationship between physical activity and academic burnout.

A bias-corrected percentile bootstrap approach with 5,000 resamples was employed. As shown in Table 2, the total effect of physical activity on academic burnout was significant (*β* = −0.131, *p* < 0.001), indicating that higher physical activity was associated with lower academic burnout. After both mediators were included, the direct effect of physical activity on academic burnout remained significant but was reduced in magnitude (*β* = −0.119, *p* < 0.001), indicating partial mediation and supporting Hypothesis 1 (see Figure 4).

**Table 2:** A mediation model test of physical activity and academic burnout.

DV	IV	*Beta*	*t*	*R*	*R^2^*	*F*
AB	Sex	0.933^*^	1.393	0.292	0.085	47.389
	Age	1.138	3.257^**^			
	PA	−0.131^***^	−11.025^***^			
CR	Sex	−0.548^*^	−1.712	0.498	0.248	131.18
	Age	−0.356^**^	−2.553^***^			
	PA	0.104^***^	21.844^***^			
ES	Sex	−0.068^*^	−0.473	0.506	0.256	131.18
	Age	0.007^*^	0.092			
	PA	0.054^**^	18.457^***^			
AB	Sex	0.796^*^	1.206	0.341	0.116	40.129
	Age	1.001	2.908^*^			
	CR	−0.392^***^	−6.203^***^			
	ES	0.499^*^	−4.303^*^			
	PA	−0.119^***^	−8.608^***^			

PA indicates Physical Activity, AB indicates Academic Burnout, ES indicates Expressive Suppression, and CR indicates Cognitive Reappraisal. *p < 0.05, **p < 0.01, ***p < 0.001.

#### Indirect effects

In addition, physical activity positively predicted cognitive reappraisal (*β* = 0.104, *p* < 0.001) and expressive suppression (*β* = 0.054, *p* < 0.001). Cognitive reappraisal was negatively associated with academic burnout (*β* = −0.392, *p* < 0.001), whereas expressive suppression was positively associated with academic burnout (*β* = 0.499, *p* < 0.01) (see Table 2).

Bootstrap analyses further supported the significance of the indirect effects. The indirect pathway through cognitive reappraisal (PA → CR → AB) was significant (effect = −0.084, *SE* = 0.014, 95% *CI* [−0.112, −0.056]), accounting for 64.12% of the total effect. This finding suggests that cognitive reappraisal served as a beneficial mediator linking higher physical activity to lower academic burnout, supporting Hypothesis 2.

In contrast, the indirect pathway through expressive suppression (PA → ES → AB) was also significant (Effect = 0.056, *SE* = 0.013, 95% *CI* [0.032, 0.082]), accounting for 42.75% of the total effect, indicating an inconsistent indirect effect that partially attenuated the protective association between physical activity and academic burnout, supporting Hypothesis 3.

Taken together, these results suggest that physical activity may reduce academic burnout partly by enhancing adaptive emotion regulation, while the simultaneous pathway through expressive suppression may offset part of this beneficial association (Table 3).

**Table 3:** Indirect effect between physical activity and academic burnout.

Pathway	Effect	S.E	Percentage (%)	95%*CI*	
				Lower	Upper
Indirect 1: PA → CR → AB	−0.084	0.014	64.12	−0.112	−0.056
Indirect 2: PA → ES → AB	0.056	0.013	42.75	0.032	0.082
Direct effect	−0.119	0.014	–	−0.149	−0.090
Total effect	−0.131	0.011	–	−0.155	−0.108

## Discussion

The present study confirms the direct effect of physical activity on academic burnout among junior high school students, as well as its indirect protective role through cognitive reappraisal. Notably, expressive suppression showed an inconsistent indirect effect in this relationship, a finding that contrasts with the simplified view that all emotion regulation strategies function solely as mediators. Grounded in Gross’s Process Model of Emotion Regulation, this research positions physical activity as a contextual factor capable of simultaneously eliciting both adaptive (cognitive reappraisal) and maladaptive (expressive suppression) emotion regulation strategies, thereby extending the explanatory scope of the model into the domain of health behaviors.

A significant negative association was found between physical activity and academic burnout, suggesting that students with higher physical activity levels tended to report lower burnout. This finding is consistent with previous studies showing that insufficient physical activity is related to higher burnout, whereas regular or recreational physical activity may reduce stress, improve mood, and support academic functioning (Dow et al., 2025; Gaspar, 2025; Golshani et al., 2021; Martin-Rodriguez and Gonzalez-Prieto, 2025; Trott et al., 2024; Wolf and Rosenstock, 2017). Moreover, this association appeared to be partly explained by cognitive reappraisal. Students with higher physical activity levels were more likely to use this adaptive emotion regulation strategy, which may help them reinterpret academic stressors more flexibly and reduce emotional exhaustion and learning disengagement (Brockbank and Feldon, 2024; Kukla-Bartoszek and Głombik, 2024). Given the modest magnitude of the observed effects, these findings should be interpreted cautiously and within a broader ecological framework. Our findings not only support previous evidence that physical activity may facilitate more adaptive emotion regulation, but also suggest that different regulation strategies may operate through distinct pathways in this relationship, thereby highlighting the multidimensional nature of its psychological influence.

In contrast, expressive suppression reveals a counteracting pathway. Analysis suggests that physical activity may be associated with a generalized internal tendency toward emotional suppression in individuals. Those inclined to use expressive suppression not only inhibit emotional expression at the behavioral level but may also concurrently suppress internal affective thought processes, such as positive feelings derived from the exercise experience (Sikka et al., 2022). This tendency partially diminishes the positive affective experiences and psychological resource gains that physical activity should otherwise provide, thereby statistically “masking” its overall protective effect (Zhao and Gan, 2025). This mechanism offers a novel perspective for understanding variability in the psychological benefits of physical activity. Notably, this study did not find a stable association between expressive suppression and negative affect, which diverges from some conclusions in Western research. This inconsistency precisely highlights that the psychosocial significance of expressive suppression is highly context-dependent (Kraus et al., 2024). Within the Chinese cultural and educational context, which emphasizes collective harmony, academic achievement, and emotional restraint (Xie and Hutagalung, 2025), expressive suppression may not be simply equated with psychological maladaptation (Pan et al., 2025; Soto et al., 2011). To some extent, it may be perceived by individuals as a self-regulatory approach consistent with social norms. Consequently, its relationship with the affective experiences and academic outcomes of Chinese junior high school students exhibits a unique complexity distinct from that observed in Western individualistic cultural settings: while it may offset some benefits of physical activity by suppressing positive experiences (Humphrey and Bliuc, 2022), it may also be dissociated from negative affect, thus manifesting statistically as an inconsistent indirect pathway rather than a “risk mediator”.

Building upon these findings, practical interventions may benefit from a more integrated approach, shifting from merely encouraging greater physical activity to promoting “exercise with awareness.” Specifically, cognitive-emotion regulation training may be incorporated into school physical education curricula by guiding students to reinterpret physical challenges as opportunities for growth and setbacks in team activities as opportunities to develop social skills (Bosshard and Gomez, 2024). In addition, complementary mental health education may help students recognize the potential costs of habitual emotional suppression and adopt healthier forms of emotional expression (Wylie et al., 2025). Within the current Chinese educational context, such programs may be implemented through brief modular activities embedded in regular physical education classes, supported by basic teacher training to enhance scalability and sustainability (Andermo et al., 2020).

In summary, by discerning the dual role of emotion regulation—serving as both a mediator and an inconsistent indirect pathway in the relationship between physical activity and academic burnout—this study reveals the complexity of its underlying mechanisms and proposes a more integrated dual-pathway theoretical framework. However, given the modest magnitude of the observed associations, these findings should be interpreted cautiously within a broader ecological context involving individual, family, and school-related factors (Wang and Degol, 2015). This not only offers a novel perspective for understanding the intricate relationship between adolescent health behaviors and psychological adaptation but also provides a useful theoretical basis and identifies practical entry points for developing future evidence-based, holistic health promotion programs tailored to the specific educational and cultural context of China.

## Limitations and future directions

Several limitations should be acknowledged when interpreting the present findings. First, the cross-sectional design precludes firm causal inferences regarding the relationships among physical activity, emotion regulation, and academic burnout. Although the proposed mediation pathways were statistically supported, they should be understood as theory-driven associations rather than definitive causal mechanisms. Second, the participants in the present study were recruited from a single junior high school in Huaihua City, Hunan Province, China. Therefore, the sample may not be fully representative of adolescents from other regions, school settings, or sociocultural backgrounds, which may limit the generalizability of the present findings. Third, the observed effect sizes were relatively modest. Accordingly, although the associations were statistically significant, their practical magnitude should be interpreted cautiously within a broader ecological framework involving family, school, and individual influences. Fourth, all variables were assessed using self-report questionnaires, which may be subject to recall bias, social desirability bias, and common method variance despite the absence of serious common method bias in the present analyses.

Future research should further clarify the dynamic relationships among physical activity, emotion regulation, and academic burnout using longitudinal, experimental, or school-based intervention designs. Such approaches would help determine whether increasing physical activity can prospectively reduce burnout through improvements in adaptive emotion regulation. Moreover, future studies are encouraged to recruit more diverse samples from multiple schools and geographic regions to improve external validity and strengthen the broader applicability of the findings. In addition, future research may benefit from incorporating objective indicators of physical activity, multi-informant assessments, and broader psychosocial variables such as family support, academic climate, and peer relationships. Furthermore, future studies should examine contextual moderators, such as school climate and family support, to better understand for whom and under what conditions physical activity may be most beneficial. From an applied perspective, future interventions may combine physical activity promotion with emotion regulation training, particularly by strengthening cognitive reappraisal while reducing excessive reliance on expressive suppression, to develop more comprehensive strategies for preventing academic burnout among adolescents. Finally, future studies should also investigate the cumulative and long-term practical impact of sustained physical activity participation on academic burnout trajectories.

## Conclusion

This study provides preliminary evidence regarding the relationships among physical activity, emotion regulation, and academic burnout among junior high school students. Consistent with Hypothesis 1, physical activity was negatively associated with academic burnout, indicating that students with higher levels of physical activity tended to report lower burnout. Consistent with Hypothesis 2, cognitive reappraisal significantly mediated this association, suggesting that physical activity may be linked to lower academic burnout partly through greater use of adaptive emotion regulation strategies. In line with Hypothesis 3, expressive suppression demonstrated an inconsistent indirect effect that attenuated part of the protective association between physical activity and academic burnout. Given the modest magnitude of the observed effects, these findings should be interpreted cautiously and within a broader ecological framework. Our findings not only support previous evidence that physical activity may facilitate more adaptive emotion regulation, but also suggest that different regulation strategies may operate through distinct pathways in this relationship, thereby highlighting the multidimensional nature of its psychological influence.

## Data Availability

The original contributions presented in the study are included in the article/supplementary material, further inquiries can be directed to the corresponding authors.
